# Changing the patterns of hospitalized diabetic foot ulcer (DFU) over a 5-year period in a multi-disciplinary setting in Thailand

**DOI:** 10.1186/s12902-020-00568-7

**Published:** 2020-06-22

**Authors:** Yotsapon Thewjitcharoen, Jeeraphan Sripatpong, Sirinate Krittiyawong, Sriurai Porramatikul, Taweesak Srikummoon, Somkiet Mahaudomporn, Siriwan Butadej, Soontaree Nakasatien, Thep Himathongkam

**Affiliations:** Diabetes and Thyroid Center, Theptarin Hospital, Bangkok, Thailand

**Keywords:** Diabetic foot ulcer, Hospitalized, Trends, Thailand, PAD

## Abstract

**Background:**

After years of decline, the rate of amputations was reported to increase by 50% in the U.S. population between 2009 and 2015. Few studies have examined the most recent trends in hospitalized diabetic foot ulcer (DFU) in Asian patients. This study aimed to examine recent trends and outcomes in hospitalized DFU at a tertiary diabetes center in Bangkok.

**Methods:**

We conducted a retrospective study from consecutive hospitalized DFU admissions from 2014 to 2018 at Theptarin Hospital, a multi-disciplinary diabetes center, led by diabetologists.

**Results:**

During the study period, 290 patients (male 57.4%, age 65.5 ± 13.3 years, T2DM 99.4%, DM duration 18.8 ± 11.5 years, A1C 8.6 ± 2.3%) with 350 admissions were included. DFU were classified into neuropathic wounds (38.0%), ischemic wounds (2.6%), and mixed-type wounds (59.4%). The median length of stay was 8 days. Severe DFU (Wagner grade 3–5) composed 68.3% of all DFU and one-third of patients had prior history of amputations. Complete healing was achieved in 73.5% of the patients. Major amputation was performed in 16 (4.6%) and minor amputation was performed in 78 (22.3%) of all DFU. The mortality rate at 1 year after discharge was 12.0%. Advanced diseases with higher co-morbidities were associated with worse outcomes. When compared with our previous published data from 2009 to 2013, the annual rate of ischemic wounds from peripheral arterial diseases (PAD) and severity of DFU were increased in this study period. The major amputation rate slightly decreased from 6.0 to 4.6% but the minor amputation rate increased from 18.7 to 22.3%.

**Conclusion:**

The changing trend of DFU provides an excellent outlook into the inadequacies of our current diabetes care systems and global trend of aging population. After considerable successes in reducing major amputations over the past decade, the current analysis revealed a discouraging change in the healing rate of DFU and a stable pattern of major amputation. The prevalence of PAD among Thai patients with DFU increased significantly and affected the results of DFU treatments. Redefined organization of care with multidisciplinary team approach and coordination with referral centers are urgently required to improve outcomes of DFU.

## Background

Despite advances in the treatments of diabetes in the last few decades, diabetic foot problems continue to be a major global burden for patients and the health care system especially in resource-limited settings [1]. Diabetic foot ulcer (DFU) only represents one aspect of multi-systemic complications of diabetes and complex co-morbidities in these patients. It also predicts increased mortality rate especially in patients with peripheral artery disease (PAD) [2–4]. Successful DFU management requires coordination among multi-disciplinary specialists to prevent foot amputations and reduce the risk of ulcer recurrences. Moreover, holistic approach should be implemented to address all of co-morbidities in DFU patients due to the presence of co-morbidities affecting the ulcer-related outcomes [5–7].

Global efforts to reduce amputation rates in DFU patients are encouraging [8]; however, the rate of amputations was reported to increase by 50% in the U.S. population between 2009 and 2015 after years of decline [9]. Whether this reversal trend could also be seen in Asian countries where infra-structure systems and required specialties like podiatrists are less developed remains unknown. Data from the United States suggested that failures in the prevention of foot ulcer and delays in timely treatment of ulcers especially in young and middle-aged people with diabetes could explain the resurgence of diabetes-related amputations [10]. Rising rates of youth-onset type 2 diabetes mellitus (T2DM) and obesity all over the world [11] might predict the grim outlook of DFU-related outcomes as seen in the United States.

Over the past 2 decades, we have increasingly been able to provide comprehensive diabetic foot care to the majority of complex patients referred to us. Our previous data showed that achievement of limb salvage rate above 90% and a complete healing rate above 80% could be possible with a dedicated team of multi-specialists [12]. But once an integrated team is built, there are always challenges and opportunities for maintaining and improving the quality of care. In contrast to the United States, diabetic foot teams in most dedicated centers have most often been led by endocrinologists in Asia and Europe [13]. The prevalence of co-morbidities such as coronary artery disease and nephropathy is high in these patients and cause increasingly difficult wounds, leading to the need for initiation of advanced wound healing treatment plans. Moreover, PAD has become a rising cause of DFU as well as affecting the healing rate and amputation-free survival [14]. Therefore, the most recent trends in hospitalized DFU in Asian patients should be explored and impacts of co-morbidities toward ulcer-related outcomes should be examined.

The primary objectives of this study were to gain a better understanding of whether the characteristics and outcomes of DFU patients have changed in the past 5 years when compared with our previous study in the last decade. The secondary objective was to evaluate the role of co-morbidities in the outcomes of DFU.

## Methods

We retrospectively reviewed medical records of all consecutive hospitalized DFU admissions from 2014 to 2018 at Theptarin Hospital, a private multi-disciplinary diabetes center in Bangkok. At our center, diabetologists take the lead role in foot care within the multidisciplinary team which consisting of general surgeons, vascular surgeons, an infectious specialist, cardiologists, diabetes nurse educators, and physical therapists. Baseline characteristics of patients including Charlson Comorbidity Index (CCI) were collected. CCI is a valid prognostic indicator for mortality within 1 year by stratifying patients into 3 categories (mild CCI 1–2; moderate CCI 3–4; and severe CCI ≥5) [15]. The presence of ischemic heart disease and/or heart failure had been grouped as having cardiovascular disease. The presence of Chronic Kidney Disease (CKD) was defined as having glomerular filtration rate less than 60 mL/min/1.73 m^2^. Date at admission was defined as the index date for follow-up. Complete wound healing was defined as the complete epithelialization of the overlying soft-tissue wound within 12 months after admission. Amputations were divided into minor (up to below the ankle level) and major amputations (above the ankle level). If a minor amputation was done and the duration of wound healing was less than 12 months, complete healing outcome was also noted. Amputation-free survival rate was defined as the percentage of patients who survived without major amputations. Patients who died before wound healing was achieved were considered to have non-healing ulcers. Causes of death were categorized into cardiovascular diseases, stroke, sepsis, cancer, and others.

In this study, both Wagner’s grading system and University of Texas system (UT classification) were used to classify the severity of ulcers. PAD was defined if distal pulses were absent and/or the ankle brachial index (ABI) was < 0.9. In patients whose ABI was > 1.4 or in those with diagnostic uncertainty, a toe pressure of < 55 mmHg or a toe brachial index (TBI) of < 0.7 was used to diagnose PAD. In patients who underwent percutaneous transluminal angioplasty (PTA), successful revascularization was defined as success in crossing the lesion with the guide-wire with or without ballooning.

The clinical data and ulcer-related outcomes in this contemporary cohort were compared with our previous published data during the period 2009–2013 (*N* = 262 ulcers) [12]. This study was approved by the Institutional Review Board (IRB) committee of Theptarin Hospital (EC No.5–2019).

### Statistical analysis

Continuous variables were presented as mean ± SD or median (IQR), as appropriate and categorical variables were presented as proportions. Comparisons between healed ulcers and non-healed ulcers were done using an unpaired Student’s t-test in continuous data and using a Chi-square test in categorical data. Kaplan–Meier survival curves and Cox proportional hazard ratios by using a forward stepwise selection method were generated for the effects of type of DFU, severity of CCI, and cardio-renal co-morbidities on ulcer healing status. Variables with established association with ulcer-related outcomes were selected for univariate analysis, and those with a *P*-value < 0.1 were included in the multivariate models to determine associated clinical factors and DFU outcomes. *P*-value < 0.05 was considered statistically significant. All statistical analyses were conducted using the Statistical Package for the Social Sciences (version 22.0; SPSS, Armonk, NY).

## Results

### Patient characteristics

**Fig. 1:**
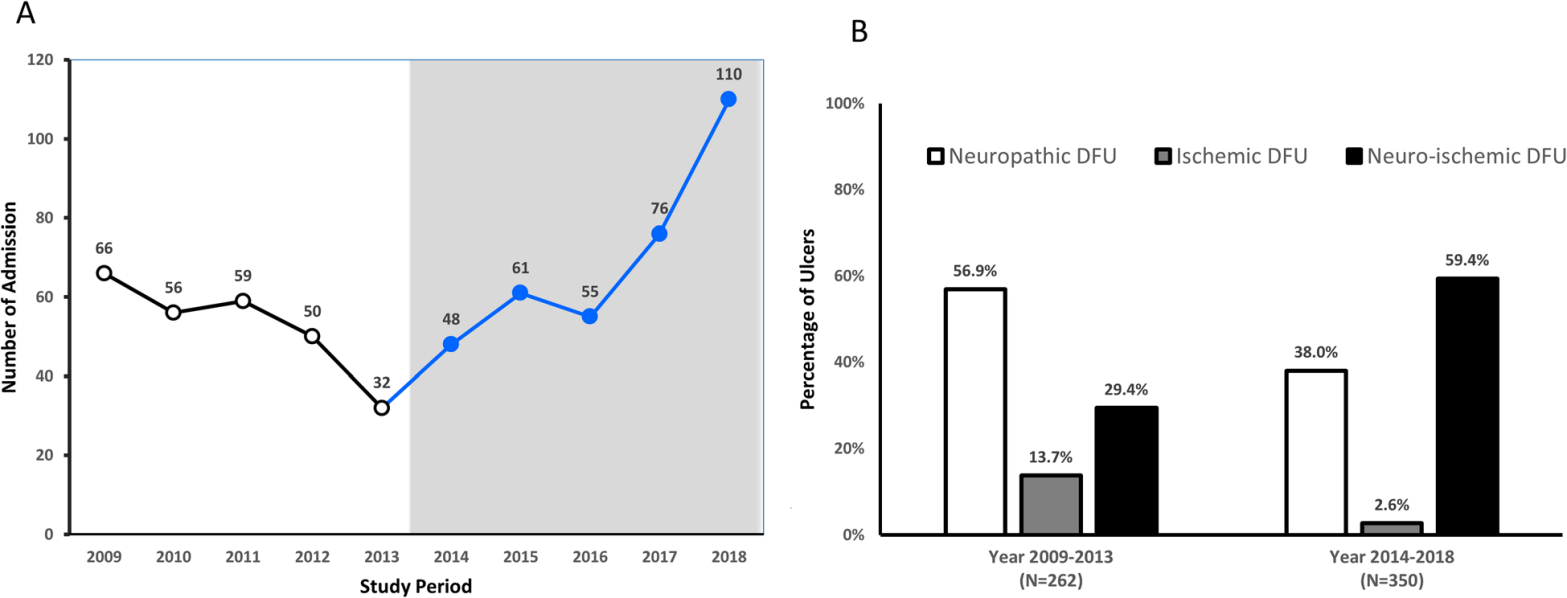
**a** The trend of hospitalized diabetic foot ulcer (DFU) from the current study period (2014–2018) compared with our previous data (2009–2013). **b** Comparison type of DFU between the current study period and previous data

During the study period (2014–2018), 290 patients (male 57.4%, age 65.5 ± 13.3 years, T2DM 99.0%, DM duration 18.8 ± 11.5 years, A1C 8.6 ± 2.3%) with 350 admissions were included. The median length of stay was 8 days (IQR 4–14 days) and the mean follow-up time was 10.7 ± 12.6 months. Diabetic foot infections were the leading causes of hospitalization (88.6%), followed by PAD which needed revascularization (9.1%), and other causes (2.3%). DFU were classified into neuropathic wounds (38.0%), ischemic wounds (2.6%), and neuro-ischemic wounds (59.4%). The distribution of the ulcers according to the Wagner’s grading system was as follows: Wagner 1 (8.6%); Wagner 2 (23.1%); Wagner 3 (62.6%); Wagner 4 (4.8%); and Wagner 5 (0.9%). The UT classification revealed UT stages C and D comprised 51.1% of the admissions. Severe DFU (Wagner grade 3–5) composed of 68.0% of all DFU and one-third of patients had prior history of amputations. The details of demographic data of DFU classified by type of ulcers had been shown in Table 1. When compared this contemporary cohort with our previous published data from 2009 to 2013, the rate of ischemic and neuro-ischemic wounds from PAD increased from 43.1 to 62.0% and severe DFU (Wagner ≥ grade 3) increased from 44.7 to 68.0%. However, the mean age of hospitalized patients was comparable between 2 periods (mean age 65.6 ± 11.9 years in earlier cohort compared with 65.5 ± 13.0 years in this current cohort). The trend of hospitalized DFU from 2009 to 2018 was depicted in Fig. 1a and b.

**Table 1:** Demographic data of diabetic foot ulcer (DFU) classified by type of ulcers during the study period (*N* = 350 admissions)

	Total	Neuropathy	Ischemia	Mixed	*p*-value
Age (years)	65.5 ± 13.3	60.2 ± 11.6	74.2 ± 12.8	68.4 ± 13.4	< 0.001
Male (%)	57.4%	59.4%	33.3%	57.2%	0.308
BMI (kg/m^2^)	25.3 ± 4.8	26.4 ± 5.0	21.5 ± 2.5	24.8 ± 4.6	< 0.001
T2DM (%)	99.4%	100.0%	100.0%	99.0%	0.503
Duration of diabetes (years)	18.8 ± 11.5	16.2 ± 10.2	13.9 ± 10.0	20.7 ± 11.9	0.001
A1C (%)	8.6 ± 2.3	9.2 ± 2.5	7.3 ± 1.9	8.2 ± 2.1	< 0.001
DR (%)	73.8%	77.8%	0%	72.4%	0.171
-NPDR (%)	15.6%	13.6%	0%	17.1%	
-PDR (%)	58.2%	64.2%	0%	55.3%	
CHF (%)	18.3%	9.8%	22.2%	23.6%	0.005
ESRD (%)	18.3%	10.5%	22.2%	23.1%	0.013
CKD (%)	57.6%	45.1%	55.6%	65.7%	0.004
Charcot (%)	10.6%	12.0%	0.0%	10.1%	0.493
IHD (%)	30.6%	11.3%	44.4%	42.3%	< 0.001
Hypertension	78.3%	66.9%	66.7%	86.1%	< 0.001
Stroke (%)	8.6%	4.5%	0.0%	11.5%	0.050
CCI ≥5 (%)	69.7%	41.4%	88.9%	87.0%	< 0.001
Wagner≥3	68.3%	54.1%	66.7%	77.4%	< 0.001
Texas stage C or D (%)	51.1%	0.8%	88.9%	81.7%	< 0.001
Ulcer Site					0.134
- Right (%)	52.9%	55.6%	88.9%	49.5%	
- Left (%)	45.4%	43.6%	11.1%	48.1%	
- Both (%)	1.7%	0.8%	0.0%	2.4%	
Previous DFU (%)	74.3%	74.4%	44.4%	75.5%	0.113
Previous Amputation (%)	32.3%	21.8%	11.1%	39.9%	0.001
Smoking (%)	30.6%	30.8%	11.1%	31.3%	0.566

Note: *p*-value indicated the differences between 3 types of DFU using one-way ANOVA for continuous data and Chi-square for categorical data to determine group differences

Abbreviations: *BMI* Body Mass Index, *T2DM* Type 2 Diabetes Mellitus, *A1C* Glycated Hemoglobin, *DR* Diabetic Retinopathy, *NPDR* Non-proliferative Diabetic Retinopathy, *PDR* Proliferative Diabetic Retinopathy, *OAD* Oral anti-diabetic drug, *CCI* Charlson Comorbidity Index, *CHF* Congestive Heart Failure, *ESRD* End-Stage Renal Disease, *IHD* Ischemic Heart Disease, *CKD* Chronic Kidney Disease

In patients with available pus or tissue culture results (70.9%), the most commonly isolated organism was mixed organisms (mainly combinations of gram-negative organisms) in 36.2%, followed by *Staphylococcus aureus* (13.1%) and *Pseudomonas spp.* (8.3%). In the gram-negative bacilli, extended-spectrum β-lactamase (ESBL) strains were found in 10.7%. In the gram-positive cocci, methicillin-resistant *Staphylococcus aureus* (MRSA) was found only in 1.7%. When compared with patients with less severe DFU (Wagner < 3), patients with more severe DFU revealed greater prevalence of ESBL strains (13.5% versus 3.5%) and more MRSA strain (4.9% versus 1.4%). When compared the present study with our previous study from 2009 to 2013, mixed organisms had been found to increase from 33.6 to 36.2%. While ESBL gram-negative bacilli strains increased from 6.5 to 10.7%, MRSA gram-positive cocci strains decreased from 3.5 to 1.7%.

**Fig. 2:**
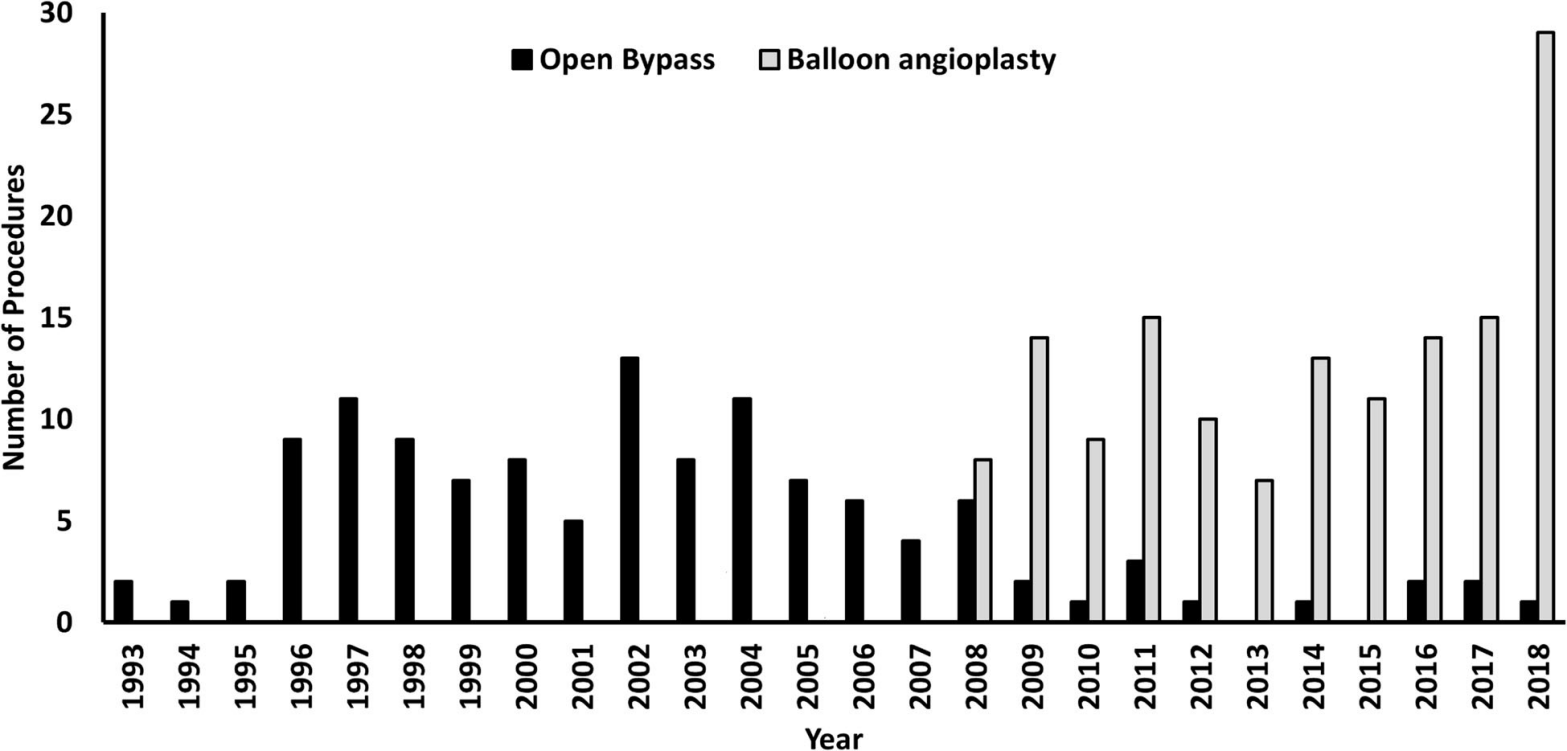
Comparison of the annual rate of revascularization procedures from 1993 to 2018 in our hospital

Among ischemic and neuro-ischemic DFU (217 ulcers), PTA was performed in 82 patients and open surgical bypass was done in only 6 patients. In our hospital, revascularization procedures have been performed with open bypass surgery since 1993 by a pioneer vascular surgeon while primary angioplasty was started in 2008 by cardiologists. As shown in Fig. 2, there has been a progressive increase in revascularization procedure by PTA from 2008. Currently, angioplasties are being performed around 20–30 cases per year when compared with open bypass surgery in only 1–2 cases per year.

### Ulcers-related outcomes

Major and minor amputations were performed in 16 (4.6%) and 78 (22.3%) of all DFU, respectively. Based on the available follow up data in 343 admissions, complete healing rate (including minor amputations) was achieved in 73.5% of the patients. The median time to heal was 85 days (range 3–365 days). The mortality rate at 1 year after discharge was 12.0%. The most common causes of death were cardiovascular events (35.5%), sepsis (21.0%), and stroke (6.5%). Ulcer-related outcomes stratified by type of ulcers are shown in Table 2. While the complete healing rate was more than 80% among neuropathic ulcers, it was only 66% in ischemic and neuro-ischemic ulcers. When compared with our previous published data from 2009 to 2013, the major amputation rate slightly increased from 4.2 to 4.6% and the minor amputation rate slight increased from 18.7 to 22.3% as shown in Table 3. The overall complete healing rate decreased significantly from 82.1 to 73.5%.

**Table 2:** Ulcer-related outcomes stratified by type of ulcers (*N* = 343 admissions)

	Total (*N* = 343)	Neuropathic wound (*n* = 130)	Ischemic wound (*n* = 9)	Neuro-ischemic wound (*n* = 204)	*p*-value
Completed healing	73.5%	85.4%	66.7%	66.2%	< 0.001
Major amputation	4.7%	0.8%	22.2%	6.4%	0.002
Minor amputation	45.5%	42.3%	22.2%	48.5%	0.196
Dead within 1 year	12.0%	5.4%	33.3%	15.2%	0.004
Recurrent DFU within 1 year^a^	44.6%	36.2%	28.6%	50.8%	0.024
Recurrent DFU within 3 years^b^	78.6%	75.0%	40.0%	82.2%	0.054

Note: *p*-value indicated the differences between 3 types of DFU using Chi-square to determine group differences

^a^ Available data in 332 admissions

^b^ Available data in 187 admissions

**Table 3:** Comparison of available ulcer-related outcomes between 2009 and 2013(*N* = 262 admissions) and 2014–2018 (*N* = 350 admissions)

	2009–2013 (*N* = 262)	2014–2018 (*N* = 350)	*p*-value
Major Amputation	4.2%	4.6%	0.783
Minor Amputation	18.7%	22.3%	0.261
Non-healing ulcer^a^	17.9%	26.5%	0.013
Dead within 1 year^a^	5.7%	12.0%	0.009

^a^ Available data in 332 admissions

### Outcomes of revascularization methods

Successful PTA procedures were achieved in 90.2% of patients who underwent the procedures. However, a total of 12 patients (16.2%) passed away after a successful PTA from underlying co-morbidities before complete wound healing. As a result, of those who had successful PTA procedures, we were able to save limb in only 46 patients (62.2%). Of the 6 patients who underwent an open bypass surgery, half of them were done the operation after failed PTA. One patient died from ischemic heart disease during hospitalization even after a successful open bypass surgery and one patient died within 1 year after the operation. As a result, the limb salvage rate was achieved only 4 patients from a total of 6 patients (66.7%).

### Associated factors with ulcer-related outcomes and amputation-free survival

**Fig. 3:**
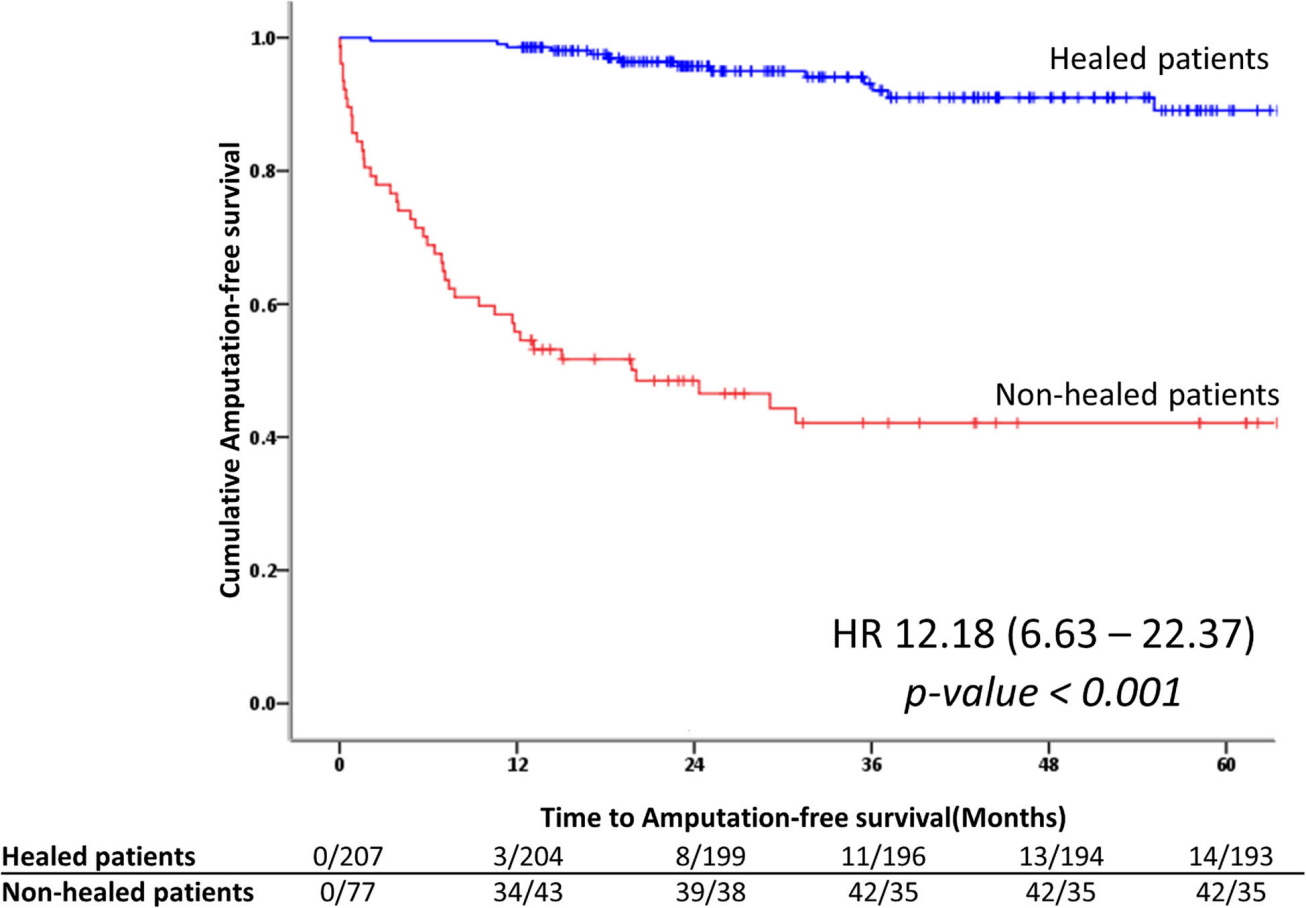
The Kaplan-Meier curves for the amputation-free survival in relationship to healing ulcers status

**Fig. 4:**
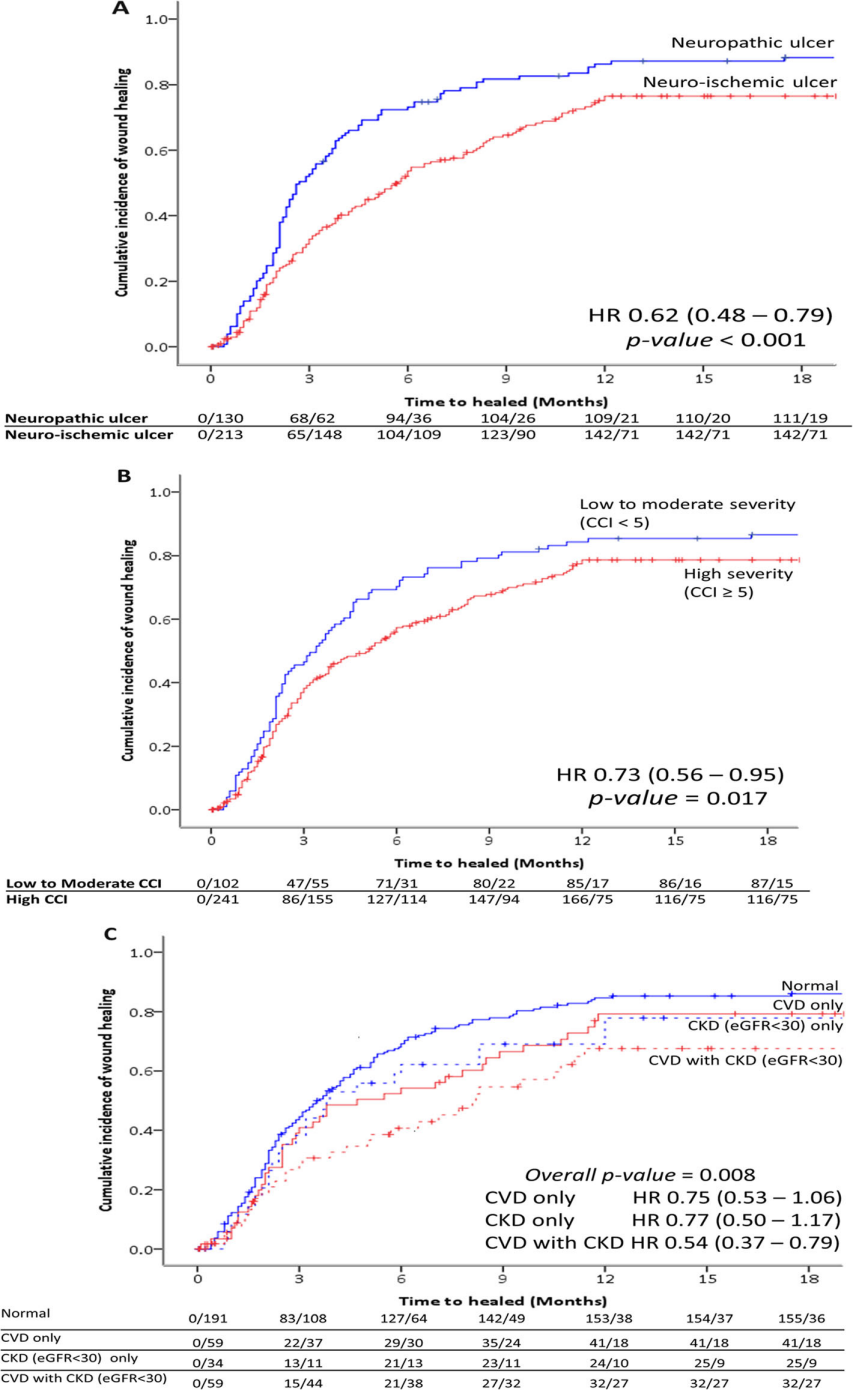
**a** The cumulative incidence of complete wound healing over time stratified by type of ulcer. **b** The cumulative incidence of complete wound healing over time stratified by severity of Charlson Co-morbidities Index (CCI). **c** The cumulative incidence of complete wound healing over time stratified by the presence or absence of cardio-renal status (CVD-Cardiovascular diseases; CKD-Chronic Kidney Disease)

According to multivariate analysis for associated factors to predict complete healing ulcers, male, BMI < 25 kg/m^2^, the presence of PAD, Wagner≥3 remained significant as shown in Table 4. The Kaplan-Meier curves for the amputation-free survival in relationship to healing ulcers status demonstrated increased survival in patients with complete healing ulcers when compared with patients with non-healing ulcers [HR 12.18, 95% CI (6.63–22.37)] as revealed in Fig. 3. The cumulative incidence of complete wound healing over time stratified by type of ulcer was statistically significantly higher in the group of neuropathic ulcers as shown in Fig. 4a. The cumulative incidence of complete wound healing over time stratified by severity of co-morbidities and the presence of cardio-renal status were demonstrated in Fig. 4b and c. Higher co-morbidity and the presence of cardio-renal status were associated with unhealed ulcers.

**Table 4:** Factors associated with complete healing diabetic foot ulcer (DFU) in the study period

Variables	Univariate			Multivariate		
	HR	***p-value***	95% CI	HR	***p-value***	95% CI
Age ≥ 60 years	1.04	0.753	0.80–1.35			
Male	0.75	0.022	0.58–0.96	0.76	0.036	0.59–0.98
BMI ≥ 25 kg/m^2^	1.48	0.002	1.16–1.90	1.39	0.010	1.08–1.80
A1C ≥ 9%	1.49	0.002	1.16–1.91	1.26	0.117	0.94–1.68
eGFR< 30 ml/1.73/m^2^	0.67	0.007	0.50–0.90	0.79	0.176	0.57–1.11
Ulcer at heel area	0.83	0.490	0.48–1.42			
PAD	0.61	< 0.001	0.48–0.79	0.71	0.025	0.53–0.96
Wagner ≥3	0.68	0.004	0.53–0.88	0.74	0.028	0.57–0.97
Texas ≥3	1.16	0.268	0.90–1.49			
CCI ≥ 5	0.73	0.018	0.56–0.95	1.16	0.404	0.82–1.64
Presence of cardiovascular diseases	0.67	0.004	0.51–0.88	0.80	0.165	0.58–1.10

## Discussions

The field of diabetes care is rapidly changing as new anti-diabetic treatments continue to emerge; however, diabetes-related chronic complications especially diabetic foot ulcers (DFU) remain a major global public health concern. DFU not only influence the patient’s quality of life, but also reduce life expectancy which can potentially be worse than some types of cancer [16]. Unfortunately, this complication had been neglected among specialists and recurrence rates are very high ranged from 30% at 1 year to almost 80% at 3 years [17]. The clinical course of DFU depends on its etiology and associated co-morbidities which some experts defined it as a ‘Diabetic Foot Syndrome’ from its complex pathogenesis and managements [6]. The problems of DFU management are not only the complexity of treatments but also inherent to the infra-structure and organization of service care. Delayed referral to multidisciplinary care and the lack of a dedicated follow-up pathway relate to poor outcomes and ultimately lead to lower extremity amputations.

In this study, our contemporary cohort showed that there had been an increase in the number of patients with complex co-morbidities and PAD which affected the outcomes of treatments. Moreover, more than three-fourths of patients previously had DFU. These changing patterns of DFU patients require a greater effort for all clinicians working in the field of diabetic foot and call for the involvement of vascular surgeons and/or interventionists in the early process of caring patients with DFU. The tertiary care centers need to be proactive in educating and coordinating with the primary care levels for early referral and an emphasis on annual screening for peripheral neuropathy and PAD in people with diabetes. In our hospital, the dedicated foot clinic service had been established since 1995 and had a uniform guideline in accordance with international standards to triage the risk of DFU in all patients with DM. For neuropathic and neuro-ischemic ulcers, the offloading techniques by various types of cast, cushioning insoles, and shoe modifications had been applied for at least 4 weeks and the wound will be re-assessed. After the hospitalized patients had been discharge, telephone follow-up will be provided by nurse educators or nurse specialists within 1 week. Our model of foot care did not change in the past decade; however, the rate of recurrent DFU is still high from patients with lost to follow-up after healed ulcers or patients who had been referred from other hospitals due to foot problems only. Therefore, coordinated cares between institutes and more efforts in healed ulcer-patients education are required to improve the future outcomes.

Diabetes is known to increase the risk for atherosclerosis including PAD which is regarded as one of established cardiovascular diseases [18]. However, the prevalence of PAD might be underestimated in people with diabetes from lack of screening and inherent limitations of ABI in calcified vessels. Moreover, neuro-ischemic diabetic foot differed from classic presentations of PAD in non-diabetic patients from its concurrent neuropathy. According to recent cross-sectional data in Chinese patients [19], PAD was highly prevalent (more than 20%) in Chinese T2DM patients but nearly half of them were undiagnosed. Several studies also suggested that people with diabetes are less likely to present with claudication symptoms [20–22]. Early detection of PAD with ABI and/or TBI screening and then initiation of optimal medical treatments is thus becoming increasingly important.

The rapid evolution of endovascular techniques globally over the last 2 decades expanded this revascularization procedure as the first choice for many centers including our hospital; however, questions over durability of patency rate, cost, and appropriate case selection remain unanswered. The Lower Extremity Guidelines Committee of the Society for Vascular Surgery (SVS) recently proposed the Lower Extremity Threatened Limb Classification System (Wound, Ischemia, foot Infection - WIfI) to predict the risk of major amputation at 1 year in a heterogeneous population of patients presenting with critical limb ischemia [23]. However, this newly proposed classification needs to validate in specific population and also whether this classification will correlate with the ulcer-related outcomes apart from major amputation require further studies. In the last decade, the European Bypass versus Angioplasty in Severe Ischemia of the Leg (BASIL) trial showed no difference in the primary endpoint of amputation-free survival between PTA and open bypass surgery [24]. However, the post-hoc analysis revealed that open bypass outperformed PTA in a subset of patients who survived for more than 2 years after procedures [25]. Currently, the Best Endovascular versus Best Surgical Therapy in patients with Critical Limb Ischemia (BEST-CLI) funded by the National Lung Heart and Blood Institute of the National Institutes of Health is recruiting over 2000 patients to answer the best approach for patients with critical limb ischemia [26]. The guideline for management of PAD in people with diabetes would also adopt the results from this mega-trial soon once the results are available in the next 4–6 years. In a real-life setting, the decision to recommend PTA or open bypass surgery has been based on not only patients’ factors but also experience from operators and availability of infra-structure resources in each center. But it should be noted that a vascular consultation should be done early in DFU patients with PAD as it had been shown that delayed vascular assessment beyond 2 weeks is associated with a lower chance to salvage the limb [27]. Moreover, based on the recent evidence, the first revascularization treatment failure seemed to affect the success of subsequent revascularizations [28, 29].

Our study also highlighted the importance of co-morbidities and ulcer-related outcomes. Due to the presence of several co-morbidities, diabetic foot patients are often very fragile subjects and foot ulceration represents only one manifestation of a complex clinical syndrome. Our findings were consistent with previous studies in Caucasian patients that co-morbidities played a key role in determining the outcomes of patients with DFU [30, 31]. These observations highlighted that DFU should be recognized as a presentation of underlying multi-organ manifestations. The landmark EURODIALE study reported that patients with heart failure and CKD patients had a greater incidence of PAD and impacted the outcomes of treatments [22]. On the other hand, patients with PAD is accompanied by ischemic heart disease in 50% of patients and more than half suffer from various degree of renal insufficiency [20]. Therefore, the impact of co-morbidities in patients with DFU should be discussed in the setting of dedicated multidisciplinary team in order to provide the best and appropriate treatments in each patient. Based on these changing trends of hospitalized DFU patients, the substantial health care and societal costs are expected to further escalate in the aging society and increasing prevalence of PAD. Redefined organization of care with multidisciplinary team approach and coordination with referral centers are urgently required to improve outcomes of DFU.

The main goal of DFU treatments should shift away from complete healing status and limb salvage to prolonged and/or avoid recurrent ulcers [32]. With accumulative data on diabetic foot treatments, all data showed consistently a very high rate of recurrent ulcers within 5 years after the development of first ulcer. Recently, experts call for ‘wound in remission’ as a more suitable surrogate endpoint than being healed in the treatment of DFU [33, 34]. Effective measures to prevent recurrent ulcers are challenging as the current available methods have not succeeded greatly in decreasing recurrent ulceration in previously DFU patients. The patients themselves are the most important member of the team in the process of foot care. The occurrence of DFU in already fragile patients could worsen their physical status and quality of life. Healthcare providers should use every opportunity to remind high-risk patients of prevention methods and incidence of new foot ulceration should be set as one of the key performance indicators of effectiveness in diabetic foot clinics. Creating a network of multidisciplinary teams who specialize in treating diabetic foot problems with a clear pathway for referral of complex DFU patients might be the best way to achieve limb salvage [35, 36]. Various diabetes guidelines repeatedly advocated that all patients should have their diabetic foot risk classification annually and receive education in foot cares. However, the annual rate of foot screening in the physician’s office is still unsatisfactory.

Our data were also consistent with other studies reporting a reduction in the rates of major amputation in DFU patients but a rise in the rates of minor amputation [8]. Even though the prevention of lower limb amputation was regarded as the successful parameter in DFU patients [37], it should be emphasized that one of the goals for DFU treatment is to maintain a functional foot and quality of life [38]. The rate of amputation is also influenced by ethnicity from some beliefs and cultures especially in the Asian countries [39]. Many patients desired to have their feet intact via a choice of conservative surgery, an option to remove some parts of infected bone and non-viable soft tissue while maintaining the external appearance of the foot even though the function of foot would be better if an amputation was done. Therefore, the concept of amputation-free survival should be balanced with quality of life in terms of ambulation and social independence. An amputation maybe a viable choice of treatment for patients who have a limited life expectancy and prolonged course of treatment is predicted to save the affected limb.

There are several limitations that should be acknowledged in this study. First, this was a retrospective study from a private setting in Bangkok with expertise in DFU for more than 2 decades. Therefore, the results may not be applicable to other populations. Economic consideration is a main obstacle for patients with low socio-economic capacity to achieve the best results. Second, the validated co-morbidities score with CCI was adopted in our study because of no DFU-specific co-morbidities score. However, the original CCI was developed in the 1980s to predict 1 year mortality in a single US hospital. In the future, more DFU-specific co-morbidities score including nutritional status assessment should be prospectively studied to refine more risk stratification. Third, the endovascular revascularization technique has been established as first choice for the correction of PAD in our study due to local preference and cost consideration. The outcomes of DFU with PAD would be different if an open bypass surgery is equally performed or has been selected based on patients’ suitability and patterns of vascular stenosis.

## Conclusions

In conclusion, the DFU provides an excellent window into the inadequacies of the current diabetes care systems and global trend of aging population. The prevalence of PAD among Thai patients with DFU increased significantly in our contemporary cohort and affected the results of treatments. The presence of complex co-morbidities particularly cardio-renal status had been associated with non-healing ulcers and increased mortality rate. Therefore, DFU should be viewed as a syndrome which requires a treatment plan for ulcers and also an evaluation of all co-morbidities that might influence the outcomes. Redefined organization of care with multidisciplinary team approach and coordination with primary care are required to improve the outcomes of DFU.

## Data Availability

The datasets supporting the conclusions of this article are publically available on reasonable request by contacting the corresponding author.

## Abbreviations

ABI: Ankle Brachial Index
CCI: Charlson Comorbidity Index
CKD: Chronic Kidney Disease
DFU: Diabetic Foot Ulcer
PAD: Peripheral Artery Disease
PTA: Percutaneous Transluminal Angioplasty
T2DM: Type 2 Diabetes Mellitus
TBI: Toe Brachial Index
UT classification: University of Texas system classification
